# Progress in Anæsthetics

**Published:** 1903-12-05

**Authors:** 


### Progress in Anæsthetics.

The special Chloroform Committee appointed by the British Medical Association has published its second report,[1] dealing chiefly with the exact dosage of chloroform required to induce and maintain narcosis. The word dosage is used to signify, not the absolute amount of the drug given, but the percentage of it which is mixed with the air which the patient inhales. The need of a method for mixing definite proportions of air and chloroform has been met to a large extent by the apparatus invented by Mr. Vernon Harcourt. The percentage of chloroform necessary varied between ·5 and 2. In a series of eight cases of narcosis in which this apparatus was employed the following were the most noticeable points: (*a*) The facility with which patients inhaled; (*b*) the slight amount of excitement or struggling; (*c*) the higher degree of the narcosis and the readiness with which it lightened; (*d*) the rapid recovery; (*e*) absence of anomalous symptoms. In reference to the clinical aspect of the question of exact dosage Dudley Buxton suggests that the intermittence of delivery from a Junker apparatus is a source of danger, and that in the case of excited or refractory patients the temptation to compress the bellows too vigorously has too often been the cause of fatal results. The more ideal method of administering the drug is one which presents to the patient a uniform mixture of chloroform and air, as does the apparatus introduced by Mr. Vernon Harcourt. The effect of chloroform upon the heart muscle has been investigated by Professor Sherrington and Miss Sowton, whose experiments show that when Ringer's solution, containing a definite percentage of $CHCl_3$, is made to circulate through the coronary vessels of isolated hearts (cats') the paralysing effects are not cumulative when weak solutions are used, and that the degree of paralysis is directly proportionate to the percentage of chloroform in the circulating fluid.

But that other factors than mere dosage have a share in the causation of mishaps is well known, and Dr. McCardie [2] lays great stress upon the number of instances in which dangerous symptoms and even deaths have been attributable to sheer nervousness or horror of the anæsthetic: he quotes one case where the patient, a boy, ceased to breathe and became pulseless while the chloroform was being brought from the adjoining room. Recovery took place after the usual means of resuscitation had been undertaken. It transpired that the boy had cried bitterly for hours on learning that he was to undergo an operation. As McCardie remarks, had a few whiffs even been given the condition would have been ascribed to that, and another death under chloroform would have been recorded. In two fatal cases under Bickle's knowledge—one under chloroform the other under ether—the patient in each case had previously declared a foreboding that the anæsthetic would be fatal.

Suggestions are thrown out by McCardie as to means by which to avoid, as far as possible, upsetting the patient's state of mind before an operation. For instance, he might be spared a longer period of anticipation than is absolutely necessary. In many cases it would be an advantage to the anæsthetist if he were introduced to his patient a day or so before the operation, because "some people have a horror of putting their lives in the hands of an utter stranger." Apropos of restoratives, McCardie thinks that the therapeutic properties of atropine are not sufficiently taken advantage of by anæsthetists. The efficacy of this drug has been proved by vivisection experiments and has been further testified to by cases of anæsthetic syncope in human beings where the administration of the drug has been followed by return of the heart-beat and recovery of the patient.

There have recently been recorded some remarkable instances of recovery after prolonged periods of cessation of breathing while under anæsthetics. One case related by Dr. David Lamb,[3] was that of a well-built man aged 26, who, as discovered post-mortem, was the subject of a cerebellar abscess. Breathing ceased when the patient was lifted from the anæsthetic couch on to the operation table. Artificial respiration and the usual methods of resuscitation having failed to establish voluntary breathing, tracheotomy was performed, and oxygen was administered through the tracheotomy tube, and the operation of trephining was proceeded with. There being no evidence of cerebral abscess the wound was closed, but in the meantime the respiratory muscles had responded to electrical stimulus and respiration now became voluntary. Thirty hours later a similar respiratory spasm occurred, and treatment, including the application of the faradic current, was kept up for two hours, at the end of which time the patient died.

In another case, recorded by Dr. H. H. Everett,[4] recovery occurred after artificial respiration had been maintained for 4 hours and 20 minutes; the case is especially remarkable owing to the fact that adrenalin chloride is credited with causing its successful termination. The patient was a male aged 20 years, the subject of acute gangrenous appendicitis, with general peritonitis, and a collection of pus in the lower part of the abdomen. During the flushing of the peritoneal cavity with saline solution there was first noticed some indication of respiratory embarrassment, ether was discontinued, and strychnine $\frac{1}{20}$ grain was given hypodermically together with solution of adrenalin chloride ♏30 by mouth. The respirations, however, became progressively slower, and in 10 minutes ceased. The pulse dropped from 120 to 96, where it remained. Artificial respiration was continued for 4 hours and 20 minutes before the slightest sign of returning voluntary breathing became apparent. During this time many expedients were tried—including strychnine, atropine, and adrenalin chloride. The latter was given in two doses of 30 minims each and was then repeated in 10 minim doses every hour.

Dr. Guthrie[5] has written at some length on some cases in which death occurred at intervals varying from ten hours to six days after the anæsthetic, and was apparently attributable to poisoning by chloroform. In none of his cases was there anything unusual noticed in the anæsthesia or in the immediate recovery from its effects, but after an interval of some hours, symptoms, generally of a cerebral type, came on; there were restlessness, screaming, and often persistent vomiting, the vomited matter resembling the dregs of beef tea. The temperature was often fluctuating and was generally above normal at the time of death, which in most cases occurred suddenly from heart failure. Jaundice was frequent. Post-mortem, the most constant feature was a peculiar fatty condition of the liver "the organ was of a uniform pale fawn or buff colour, studded internally with pale purple dots, which were the intralobular veins. Fatty livers are common enough, . . . but the pale light fawn colour of the liver in these cases was altogether unusual." The microscope revealed a condition of extreme fatty infiltration. The suggested explanation is that the liver in such cases is fatty before the operation is undertaken, and that this condition is aggravated by the combined effects of the anæsthetic and the operation shock, so that the system becomes loaded with toxic alkaloids. The counter suggestions are that death may be due to carbolic acid poisoning or to fat embolism. As regards carbolic acid, it was used as a matter of routine in all of Dr. Guthrie's cases, but in one case, a typical one of the series, it was only used in a 1 in 40 solution to wash the skin before operation, and was immediately washed off with perchloride of mercury solution. Moreover, carboluria did not occur in this patient. As to fat embolism—its presence was disproved by microscopic examination of the lungs and brain.

It is interesting to see that ethyl chloride has found a champion in such an authority as Dr. McCardie,[6] who advocates this anæsthetic for such cases as tonsils and adenoids and for operations of not more than 15 or 20 minutes' duration. He says that he now gives it with confidence, and out of a total of more than 1,000 cases last year of administrations in general, as apart from dental surgery, he administered it no fewer than 350 times. McCardie thinks that the accounts of excitement recorded during ethyl chloride anæsthesia and the failures to induce narcosis are due to too much mingling of air with the vapour. He himself has been able to anæsthetize successfully with ethyl chloride patients (strong men) with whom nitrous oxide had previously failed.

The introduction of a new anæsthetic is necessarily followed by the invention of new contrivances by which to administer it. By a simple modification T. D. Luke[7] has adopted the bag and face-piece of an ordinary Clover's inhaler for use with ethyl chloride. A hole is bored on the upper side of the horizontal limb of the elbow tube (joining the bag to the face-piece), and in this a narrow tube, about $\frac{3}{16}$-inch in diameter and $1\frac{1}{2}$ inch long, is fixed so as to pass down in the lumen of the larger tube into the bag, and through this fine tube the ethyl chloride is sprayed into the bag. The bag is fitted on to the face-piece by means of the elbow tube without the intervention of the ether chamber. To prevent escape of the vapour a small wooden plug is fitted to the end of the narrow tube.

H. Bellamy Gardner[8] has brought out a new apparatus for giving gas and ether. It consists of a face-piece attached by means of a stopcock to an ether bag, with a sponge cage interposed between the bag and the stopcock. To that side of the stopcock which is opposite to the face-piece there can be attached a second bag for gas—the gas bag is supplied with valves.

G. B. Flux[9] advocates the "open" method of administering nitrous oxide, and for this purpose has invented an apparatus by means of which a mixture of nitrous oxide and air is delivered to the patient. The chief feature of the instrument is that the supply of air is constant and is not controlled by any tap, whereas the amount of gas given is easily regulated.

[1] Supplement to Brit. Med. Jour., July 18, 1903. [2] Birmingham Med. Rev., March, 1903. [3] Lancet, May 16, 1903. [4] Med. Rec., May 23, 1903. [5] Lancet, July 4, 1903. [6] Ibid., April 4, 1903. [7] Ibid., July 18, 1903. [8] Ibid. [9] Ibid., April 4, 1903.

